# Kinetic Modification on Hydrogen Desorption of Lithium Hydride and Magnesium Amide System

**DOI:** 10.3390/ma8073896

**Published:** 2015-06-29

**Authors:** Hiroki Miyaoka, Yongming Wang, Satoshi Hino, Shigehito Isobe, Kazuhiko Tokoyoda, Takayuki Ichikawa, Yoshitsugu Kojima

**Affiliations:** 1 Institute for Sustainable Sciences and Development, Hiroshima University, 1-3-1 Kagamiyama, Higashi-Hiroshima 739-8530, Japan; 2 Creative Research Institution, Hokkaido University, N-21, W-10, Sapporo 001-0021, Japan; E-Mails: wang@eng.hokudai.ac.jp (Y.W.); s.hino@eng.hokudai.ac.jp (S.H.); isobe@eng.hokudai.ac.jp (S.I.); 3 Central Research Laboratory, Taiheiyo Cement Corporation, 2-4-2 Osaku, Sakura 285-8655, Japan; E-Mail: Kazuhiko_Tokoyoda@taiheiyo-cement.co.jp; 4 Graduate School of Integrated Arts and Sciences, Hiroshima University, 1-7-1 Kagamiyama, Higashi-Hiroshima 739-8521, Japan; E-Mail: tichi@hiroshima-u.ac.jp; 5 Institute for Advanced Materials Research, Hiroshima University, 1-3-1 Kagamiyama, Higashi-Hiroshima 739-8530, Japan; E-Mail: kojimay@hiroshima-u.ac.jp

**Keywords:** hydrogen storage, kinetics, crystallinity, ball-milling, mechanochemical process, lithium, magnesium

## Abstract

Various synthesis and rehydrogenation processes of lithium hydride (LiH) and magnesium amide (Mg(NH_2_)_2_) system with 8:3 molar ratio are investigated to understand the kinetic factors and effectively utilize the essential hydrogen desorption properties. For the hydrogen desorption with a solid-solid reaction, it is expected that the kinetic properties become worse by the sintering and phase separation. In fact, it is experimentally found that the low crystalline size and the close contact of LiH and Mg(NH_2_)_2_ lead to the fast hydrogen desorption. To preserve the potential hydrogen desorption properties, thermochemical and mechanochemical rehydrogenation processes are investigated. Although the only thermochemical process results in slowing the reaction rate due to the crystallization, the ball-milling can recover the original hydrogen desorption properties. Furthermore, the mechanochemical process at 150 °C is useful as the rehydrogenation technique to preserve the suitable crystalline size and mixing state of the reactants. As a result, it is demonstrated that the 8LiH and 3Mg(NH_2_)_2_ system is recognized as the potential hydrogen storage material to desorb more than 5.5 mass% of H_2_ at 150 °C.

## 1. Introduction

Hydrogen (H_2_) is an attractive energy carrier to effectively utilize natural energy resources such as solar, hydro, and wind energy. However, it is difficult to produce the high condensation state because H_2_ is in the gaseous phase under ambient conditions and the critical point is −240 °C. Thus, hydrogen storage techniques are necessary to compactly store and transport hydrogen and have been investigated. Hydrogen storage in materials is one of attractive techniques because they can store hydrogen as atomic state, resulting in the highly compact state. In 2007, a target of hydrogen storage materials for fuel cell vehicle is determined by New Energy and Industrial Technology Development Organization (NEDO) in Japan [1]. In this policy, more than 5.5 mass% of the reversible hydrogen capacity and less than 150 °C for the operating temperature are required, where these values are based on material. To achieve the above targets, the hydrogen storage materials based on light elements, lithium (Li), sodium (Na), magnesium (Mg), boron (B), carbon (C), and nitrogen (N), attract much interest because these materials can realize the high gravimetric hydrogen density, which is potentially higher than the target value [2,3,4,5,6,7,8,9,10,11]. Among them, the amide-imide system is recognized as an attractive hydrogen storage system. The reaction of typical Li system is described as follows [12]:

(1) LiH + LiNH_2_ ↔ Li_2_NH + H_2_

in this system, lithium amide (LiNH_2_) phase in the hydrogenated state is changed to imide phase (Li_2_NH) after the hydrogen desorption. Although about 6.0 mass% of hydrogen is reversibly stored, the hydrogen desorption requires more than 200 °C due to the high enthalpy change, Δ*H* = 67 kJ·mol^−1^ H_2_ [13,14,15]. To decrease the reaction temperature, the lithium hydride (LiH) and magnesium amide (Mg(NH_2_)_2_) system has been proposed. Systems with different molar ratio were reported at almost the same time by Luo *et al.* [16], Leng *et al.* [17], Xiong *et al.* [18], and Nakamori *et al.* [19]. The representative three reactions are described as follows:

(2) 6LiH + 3Mg(NH_2_)_2_ → 3Li_2_Mg(NH)_2_ + 6H_2_ (5.5 mass%)

(3) 8LiH + 3Mg(NH_2_)_2_ → 4Li_2_NH + Mg_3_N_2_ + 8H_2_ (6.9 mass%)

(4) 12LiH + 3Mg(NH_2_)_2_ → 4Li_3_N + Mg_3_N_2_ + 12H_2_ (9.1 mass%)

the detailed reaction process of the 8LiH-3Mg(NH_2_)_2_ system below 200 °C is expressed as follows [20]:

(5) 8LiH + 3Mg(NH_2_)_2_ → 3LiMgN_2_H_3_ + 5LiH + 3H_2_

(6) 8LiH + 3Mg(NH_2_)_2_ → 3Li_1+*x*_MgN_2_H_3-*x*_ + (5-3*x*)LiH + (3 + 3*x*)H_2_

(7) 8LiH + 3Mg(NH_2_)_2_ → 3Li_2.7_MgN_2_H_1.3_ + 8H_2_

where *x*: 0 < *x* < 1.7. First, 3 mol of H_2_ is desorbed by Equation (5) with the flat plateau in the pressure-composition isotherm (PCI) [21]. After that, 3LiMgN_2_H_3_ (=3LiNH_2_-3MgNH) continuously reacts with LiH and desorbs H_2_. The compositions of Li and H in the product defined as Li_1+*x*_MgN_2_H_3-*x*_ are non-stoichiometrically varied in this process, resulting in the slope in the PCI [21,22]. Under vacuum condition at 200 °C, a single phase of 3Li_2.7_MgN_2_H_1.3_ (=4Li_2_NH-Mg_3_N_2_) is finally formed, and then total 6.9 mass% of hydrogen can be desorbed [23], where this single phase would disproportionate into Li_2_NH and Mg_3_N_2_ under higher temperature condition [17]. Thus, this system is recognized as a potential hydrogen storage material to achieve the above practical properties. However, improvement of the hydrogen storage properties is still necessary. The cyclic hydrogen absorption and desorption properties were investigated by Ikeda *et al.* [24]. They reported that the initial hydrogen desorption capacity was 4.6 mass% and decayed to 3.6 mass% after 300 cycles at 200 °C. They claimed that the NH_3_ emission during the dehydrogenation is the origin of decrease in the hydrogen capacity. On the other hand, the detailed properties at 150 °C, as the target temperature, have not been clarified yet. At lower temperatures, the kinetic control would be main issue to utilize the essential hydrogen storage properties because hydrogen is desorbed by the solid-solid reaction and the hydrogen absorption proceeds with the phase separation. Namely, the crystalline size of solid materials and the contact between two solid phases would be important factors to realize suitable reaction rate at 150 °C. Furthermore, it is expected that the sintering and/or crystallization of solid materials by heating for the hydrogen desorption and absorption processes slows the reaction rates.

In this work, the various synthesis and rehydrogenation processes of the 8LiH-3Mg(NH_2_)_2_ system are investigated to understand the essential hydrogen storage properties as fundamental research. From the obtained experimental results, the feasibility of achieving 5.5 mass% H_2_ desorption at 150 °C is discussed.

## 2. Results and Discussion

**Figure 1:**
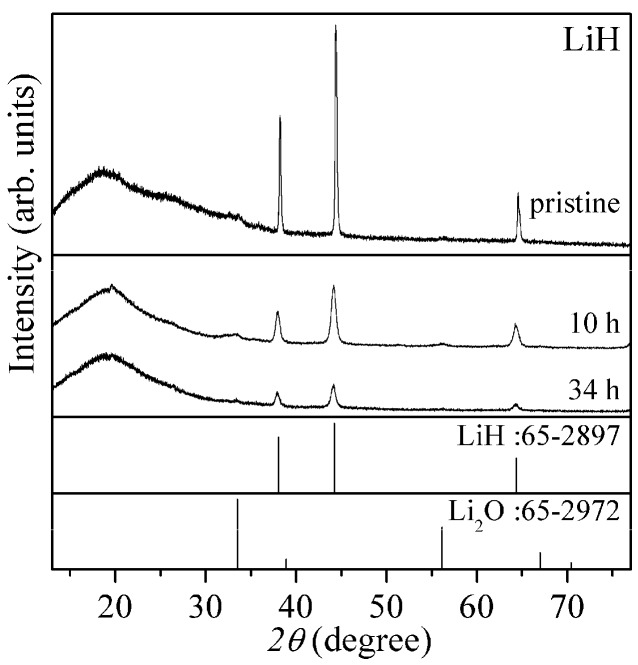
XRD (X-ray diffraction) patterns of the pristine and ball-milled LiH (10 h and 34 h). XRD patterns of LiH and Li_2_O in database are also shown as reference.

Figure 1 shows X-ray diffraction (XRD) patterns of the pristine and ball-milled LiH with the patterns of LiH and Li_2_O in database as reference. Here, broad peaks around 20° and 25° are caused by a grease to spread the powder sample and a polyimide sheet to cover the sample for avoiding the oxidation.

The diffraction peaks observed in the case of pristine LiH were high intensity and sharp. After the ball-milling as pre-treatment, the peaks were clearly lowered and broadened, suggesting that the crystalline size was reduced and the structural disorder was induced. The milling effects became slightly strong for 34 h.

**Figure 2:**
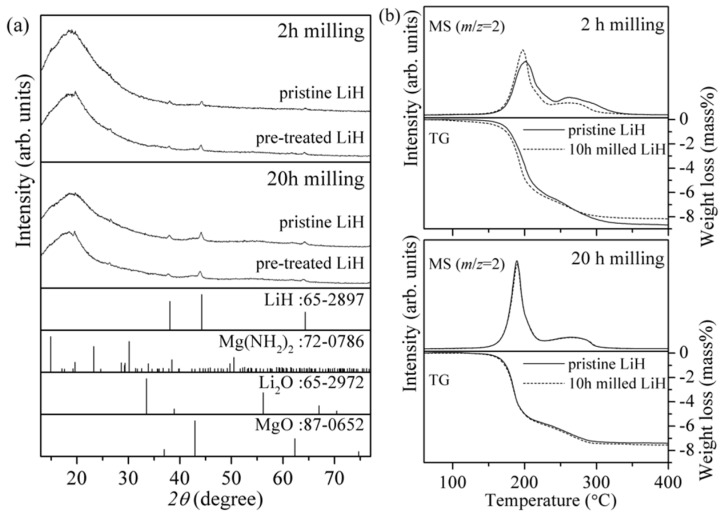
(**a**) XRD patterns and (**b**) TG-MS (thermogravimetry-mass spectroscopy) profiles of the 8LiH-3Mg(NH_2_)_2_ samples synthesized from the pristine or pre-treated LiH (10 h milling) by ball-milling for 2 h and 20 h. XRD patterns of LiH, Mg(NH_2_)_2_, Li_2_O, and MgO in database are shown as reference.

Mg(NH_2_)_2_ used in this work was synthesized by the method reported before [15,25]. Mg(NH_2_)_2_ and 10 h milled LiH were mixed with a 3:8 molar ratio by ball-milling for 2 and 20 h, and the property of each mixture was compared with the samples synthesized from the pristine LiH (see also Figure S1). Figure 2a shows XRD patterns of 2 and 20 h milled mixtures using the pristine and pretreated LiH (10 h milled). The only LiH phase was observed without impurity phases such as oxides and the peak intensity and shape were almost similar for all the samples. Mg(NH_2_)_2_ should be of nano-structure or amorphous, as no diffraction peaks appeared. From the XRD results, clear difference was not found. On the other hand, the hydrogen desorption profiles obtained by thermogravimetry-mass spectroscopy (TG-MS) showed the different behaviors as shown in Figure 2b. Here, the hydrogen desorption as shoulder around 250–300 °C would be caused by the variation of reaction path from Equations (3) to (6) with the phase separation [17]. The hydrogen desorption peaks corresponding to the samples milled for 2 h were slightly broad and weak compared with those of the sample milled for 20 h. Moreover, the weight loss corresponding to the 20 h milled sample was closer to the theoretical value, 6.9 mass%. As described above, it is known that the small amount of NH_3_ originated in the decomposition of Mg(NH_2_)_2_ is released with the H_2_ desorption. When the mixing state between LiH and Mg(NH_2_)_2_ is poor, the NH_3_ desorption amount should be increased. Thus, these results indicate that the mixing state between two solid phases started by the pre-treated LiH was much better in the 2 h milled mixtures. However, for the 20 h milled mixtures, the hydrogen desorption profile was clearly sharpened, suggesting that the two materials with the better mixing state easily reacted. In this case, the effect of pre-treatment was not clear because of the same TG-MS profiles in our experimental accuracy. It is expected that the crystalline size of LiH is reduced enough to form the good contact between LiH and Mg(NH_2_)_2_ during the 20 h milling. The weight loss corresponding to the hydrogen desorption was almost consistent with the theoretical value due to the faster reaction rate. Although it is clarified that the pre-treatment of LiH is effective to improve the kinetics of hydrogen desorption, it is not necessary when the mixing time is long enough.

**Figure 3:**
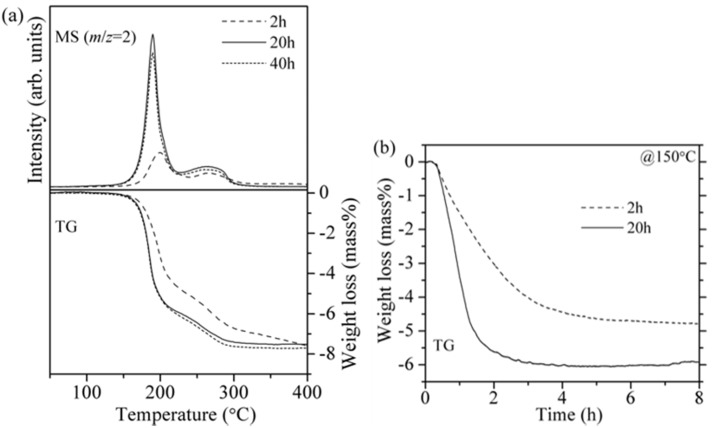
(**a**) TG-MS profiles of the 8LiH-3Mg(NH_2_)_2_ samples synthesized by ball-milling for 2, 20, and 40 h; (**b**) Isothermal TG profiles at 150 °C of the 2 h and 20 h milled samples.

In order to examine an effect of the longer mixing time of LiH and Mg(NH_2_)_2_, the sample was prepared by milling for 40 h. In Figure 3a, the TG-MS results are compared, in which LiH used here was not pre-treated. The reaction rate of the 20 h milled sample was obviously faster than that of the 2 h milled sample. However, no clear difference between the samples prepared by the 20 h and 40 h milling was found in the TG-MS profiles, indicating that the reduction of crystalline size was saturated and the close contact of two materials was realized by milling for 20 h.

Figure 3b shows isothermal TG profiles of the 2 and 20 h milled 8LiH-3Mg(NH_2_)_2_ samples at 150 °C for 8 h. For the 2 h milled sample, the weight loss due to the hydrogen desorption gradually proceeded and the hydrogen desorption amount was less than 5.0 mass%, even after 8 h. On the other hand, the 20 h milled sample revealed faster reaction rate, and then the hydrogen desorption amount reached to about 6.0 mass% within 3 h. To accurately evaluate the potential amount of hydrogen desorption, the NH_3_ emission should be considered. In previous reports, about 0.05 mol% of NH_3_ was essentially released during all the hydrogen absorption and desorption cycles at 200 °C [24]. In fact, the small amount of NH_3_ was also observed in MS measurement around 200 °C [26]. On the other hand, the MS signal corresponding to NH_3_ was quite low intensity and out of an apparatus resolution at 150 °C. Therefore, the contribution of NH_3_ emission to the weight loss in the isothermal TG measurement at 150 °C performed in this work was negligible.

**Figure 4:**
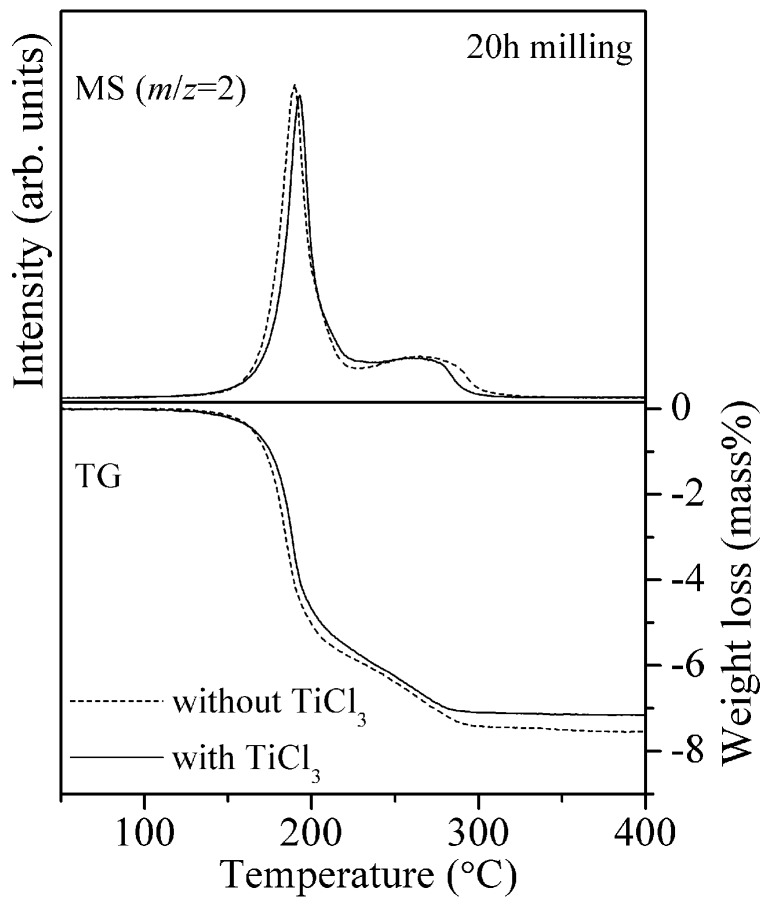
TG-MS profiles of the 8LiH-3Mg(NH_2_)_2_ samples with and without 0.54 mol% TiCl_3_ synthesized by the ball-milling for 20 h.

For the LiH-LiNH_2_ system, 1 mol% titanium chloride (TiCl_3_) shows excellent catalytic effect and lowers the peak temperature of hydrogen desorption in the MS analysis [27,28]. On the basis of previous works, the effects of TiCl_3_ on the 8LiH-3Mg(NH_2_)_2_ system was investigated. Figure 4 shows the results of TG-MS measurement for the 8LiH-3Mg(NH_2_)_2_ samples with and without TiCl_3_. The peak temperature of hydrogen desorption was located around 190 °C for both samples, and the profiles were almost same. The TG profiles were also similar, where the slight difference of weight loss at 400 °C would be caused by the TiCl_3_ addition. Thus, it was clarified that TiCl_3_ has no significant catalytic effects on the 8LiH-3Mg(NH_2_)_2_ system, suggesting that the reaction processes are possibly different from the LiH and LiNH_2_ system.

**Figure 5:**
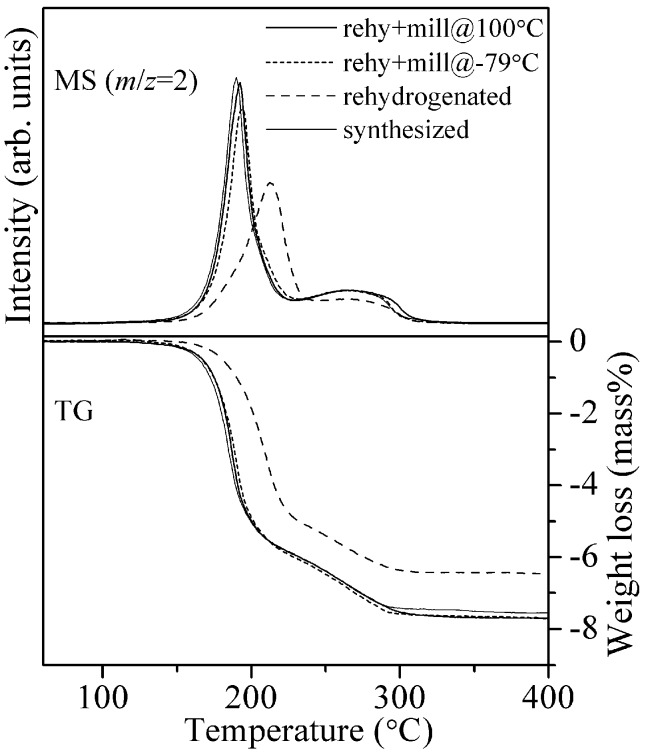
TG-MS profiles of the 8LiH-3Mg(NH_2_)_2_ samples after the synthesis, the thermochemical rehydrogenation, and the ball-milling at 100 °C and −79 °C for the rehydrogenated sample.

From the above results, the 8LiH-3Mg(NH_2_)_2_ sample synthesized from the pristine LiH by ball-milling for 20 h is chosen as the starting material for experiments to investigate the rehydrogenation processes. The synthesized 8LiH-3Mg(NH_2_)_2_ was dehydrogenated at 200 °C under dynamic vacuum condition for 24 h. The dehydrogenated sample was analyzed by the TG-MS measurement to quantitatively evaluate the progress of reaction. As a result, the weight loss with heating up to 400 °C was less than 0.5 mass% (see Figure S2), suggesting that the dehydrogenation was almost completed in this condition. The rehydrogenation was performed at 200 °C under 10.0 MPa of H_2_ for 12 h. After the thermochemical rehydrogenation, it is expected that the crystallization and sintering of the solid particles are induced. To reduce the crystalline size and recover the close contact between LiH and Mg(NH_2_)_2_, the rehydrogenated sample was ball-milled under three different temperature conditions, at room temperature, 100 °C, and −79 °C, where the products were named “rehy + mill”. Here, the results of sample prepared at room temperature is omitted in the discussion below because the properties are almost same as those prepared at 100 °C and −79 °C. Figure 5 representatively shows the TG-MS profiles of the rehy + mill@100 °C and rehy + mill@ −79 °C samples with the profiles of the samples after synthesis and thermochemical rehydrogenation. In addition, all the information obtained by TG-MS measurements is listed in Table 1.

**Table 1:** Peak temperature and weight loss obtained by TG-MS to 400 °C, weight loss obtained by isothermal TG measurements at 150 °C for 8 h, and time required to desorb 5.5 mass% of hydrogen at 150 °C of each sample.

Sample	TG-MS (to 400 °C)		Isothermal TG (150 °C, 8 h)	
	Peak temperature (°C)	Weight loss (mass%)	Weight loss (mass%)	Time to 5.5 mass% (h)
Synthesized	190	7.5	6.0	1.8
Rehydrogenated	213	6.5	4.0	> 8.0
Rehy + mill@100 °C	192	7.7	5.8	3.2
Rehy + mill@–79 °C	193	7.7	5.8	2.5
Mill-rehy@150 °C	198	7.9	5.7	4.7
Mill-rehy@RT	174	4.7	–	–

The peak temperature of hydrogen desorption of the synthesized sample was located at 190 °C. After the rehydrogenation without milling treatment, the peak was significantly shifted, to a temperature higher than 200 °C. Moreover, it was noted that the weight loss was only 6.5 mass%. The decrease in hydrogen capacity for the sample rehydrogenated by the only thermochemical reaction is, possibly, caused by the following reasons. During the heating processes for de/rehydrogenation, the crystalline size of solid materials is increased by sintering, resulting in the longer diffusion distance of atoms and the poor contact between the generated hydride and amide. In this case, the unreacted parts should remain after the TG-MS measurement. As another possibility, it seemed that the rehydrogenation was not completed under the conditions. Because both the rehy + mill samples revealed better hydrogen desorption properties, where the MS and TG profiles were almost same as those of the synthesized sample, the additional milling processes under H_2_ pressure is able to recover the close contact of both materials due to the reduction of crystalline size.

**Figure 6:**
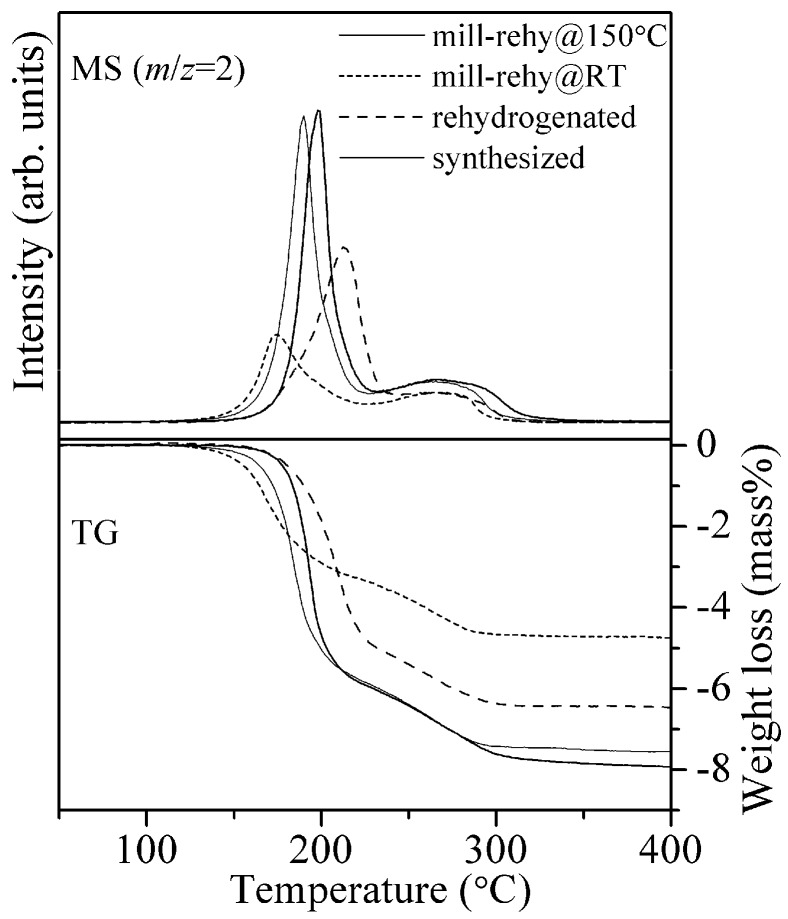
TG-MS profiles of the 8LiH-3Mg(NH_2_)_2_ samples after synthesis, thermochemical rehydrogenation, and mechanochemical rehydrogenation at room temperature and 150 °C.

The above results indicate that the small crystalline sizes should be preserved to prevent the decay of the essential hydrogen desorption properties. Thus, the rehydrogenation was performed by the mechanochemical reaction using ball-milling under H_2_ pressure, where the mechanochemically rehydrogenated samples are named “mill-rehy”. The dehydrogenated sample was milled under two conditions: room temperature under 6.0 MPa of H_2_ and 150 °C under 15.0 MPa of H_2_ for 5 h. At 150 °C, the high pressure was applied to thermodynamically suppress the hydrogen desorption. The TG-MS profiles of mill-rehy@RT and mill-rehy@150 °C are shown in Figure 6.

**Figure 7:**
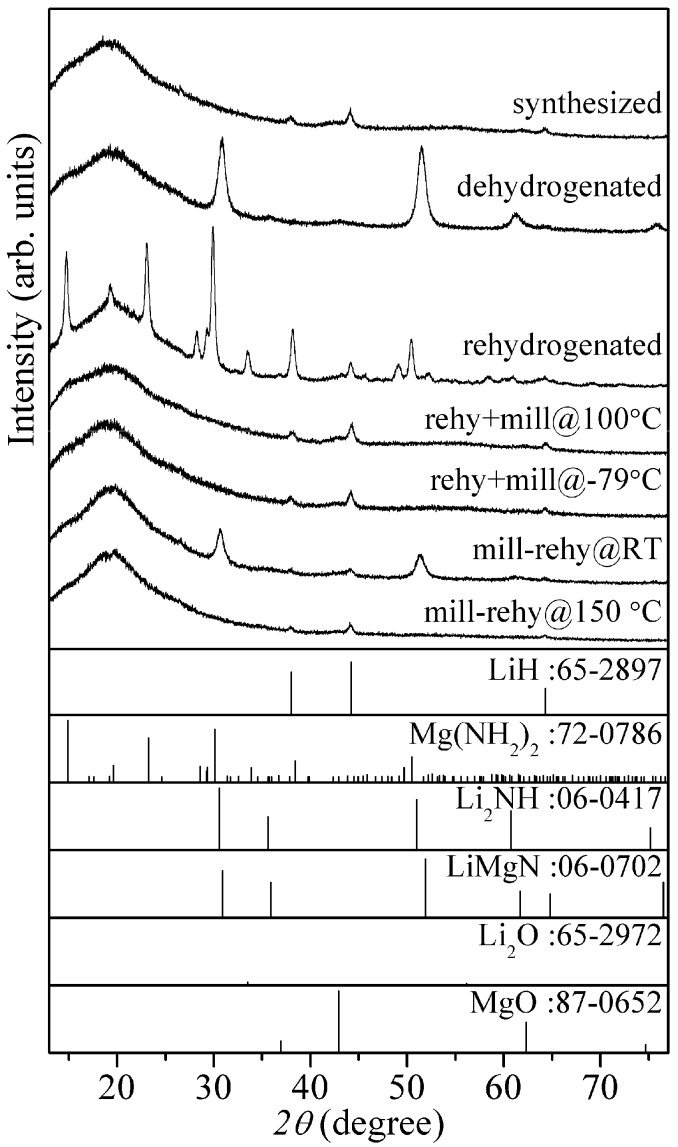
XRD patterns of the 8LiH-3Mg(NH_2_)_2_ samples after the synthesis, dehydrogenation, and various rehydrogenation processes. XRD patterns of LiH, Mg(NH_2_)_2_, Li_2_NH, LiMgN, Li_2_O, and MgO in database are shown as reference.

Figure 7 shows XRD patterns of 8LiH-3Mg(NH_2_)_2_ sample for all the processes. As already described above, the synthesized sample included the low crystalline LiH and Mg(NH_2_)_2_. After the dehydrogenation at 200 °C, the diffraction peaks were still broad shape and would be originated in the similar structure to Li_2_NH and/or LiMgN, which should be dehydrogenated state defined as Li_1+*x*_MgN_2_H_3−*x*_. In the XRD pattern of the thermochemical rehydrogenated sample, the sharp peaks corresponding to LiH and Mg(NH_2_)_2_ phases were clearly observed, suggesting that the crystallization of them proceeded during the rehydrogenation. Because the peaks assigned to the dehydrogenated phases completely disappeared, the poor hydrogen desorption properties of the rehydrogenated sample observed in the TG-MS measurement would be caused by the formation of worse mixing state between both materials due to the thermal crystallization. By the ball-milling at 100 °C and −79 °C after the thermochemical rehydrogenation, Mg(NH_2_)_2_ was changed to nano-structural or amorphous phase. The shapes of peaks corresponding to LiH were similar to those of the synthesized sample. From these results, it is indicated that the low crystalline size are recovered. In fact, the hydrogen desorption properties was improved by the milling processes as shown in Figure 5. In the XRD pattern of mill-rehy@RT, the dehydrogenated phases remained although the crystalline Mg(NH_2_)_2_ was not observed. This result is consistent with the low hydrogen desorption capacity observed in the TG-MS measurement. After the mechanochemical rehydrogenation at 150 °C, the XRD pattern was similar to the synthesized and rehy + mill samples.

**Figure 8:**
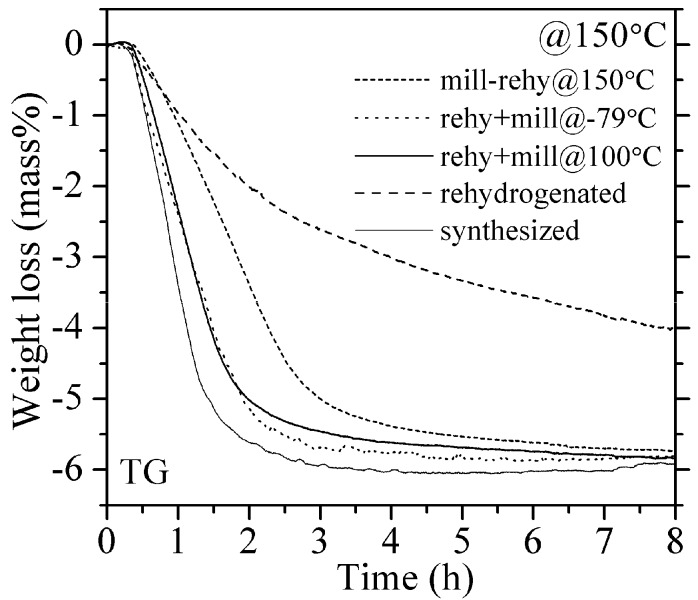
Isothermal TG profiles at 150 °C of the 8LiH-3Mg(NH_2_)_2_ samples after the synthesis, and various rehydrogenation processes.

For the samples having better hydrogenated states, rehy + mill@100 °C, rehy + mill@−79 °C, and mill-rehy@150 °C, the isothermal TG measurements were performed at 150 °C. The obtained results are shown in Figure 8 with the results of synthesized and rehydrogenated samples. Furthermore, the weight loss observed for 8 h and the time required to desorb 5.5 mass% of H_2_, which is the target value of hydrogen capacity, are listed in Table 1. The synthesized sample showed the fastest reaction rate, and about 5.5 mass% of H_2_ can be desorbed within 1.8 h. However, the hydrogen desorption of the thermochemically rehydrogenated sample was only 4.0 mass%, even after reaction for 8 h. The TG profiles of the rehy + mill samples were similar to each other and revealed relatively faster hydrogen desorption. As a result, 5.5 mass% of H_2_ was obtained within 3.2 h. Although the reaction rate of the mechanochemically rehydrogenated sample was slightly slower, the hydrogen desorption amount can reach 5.5 mass% after 4.7 h. The difference between the rehy + mill and mill-rehy samples might be caused by the temperature of the rehydrogenation processes, namely, 150 °C might be too high to realize the suitable crystalline size and mixing state by competing with the thermal crystallization. By further optimizing the treatment conditions, the hydrogen desorption properties of mechanochemically rehydrogenated sample would be improved.

All the rehydrogenated samples showed about 5.8 mass% for 8 h, although the initial reaction rate was different for each sample, and then the reaction stage should be in the non-stoichiometric variation and roughly described as follows:

(8) 8LiH + 3Mg(NH_2_)_2_ → 3Li_2.2_MgN_2_H_1.8_ + 1.6LiH + 6.4H_2_ (5.5 mass%)

(9) 8LiH + 3Mg(NH_2_)_2_ → 3Li_2.3_MgN_2_H_1.7_ + 1.3LiH + 6.7H_2_ (5.8 mass%)

from the above experimental results, it is clarified that the crystalline size and contact between LiH and Mg(NH_2_)_2_ are important factors to realize the suitable reaction kinetics. Furthermore, it is demonstrated that the 8LiH-3Mg(NH_2_)_2_ system is the potential hydrogen storage material to achieve 5.5 mass% of H_2_ desorption at 150 °C, even after the rehydrogenation by preserving the suitable crystalline size and mixing state. The reaction mechanism was proposed in previous literature, reported by Isobe *et al*. [20] and Nakamura *et al*. [23]. In the hydrogen desorption Reaction (5), H^+^ in the non-stoichiometric Li_1+*x*_MgN_2_H_3−*x*_ phase and Li^+^ in LiH are diffused and exchanged each other via thorough the interface between the solid phases, and then Li-rich/H-poor imide phase are generated. The H_2_ desorption proceeds by combining H^+^ diffused from Li_1+*x*_MgN_2_H_3−*x*_ with H^−^ in LiH. This proposed mechanism is quite reasonable from the points of view of charge and mass balance in the system. Considering the above hydrogen desorption mechanism, it is expected that the shorter diffusion distance of the ions and the larger number of interface between both materials will realize the fast hydrogen desorption. Therefore, the experimental facts in this work strongly support the reaction mechanism.

## 3. Experimental Section

Commercial LiH (99.4%, Alfa Aesar, Lancashire, UK) was used as the starting material. Mg(NH_2_)_2_ was synthesized by ball-milling MgH_2_ (95%, Gelest Inc., Morrisville, PA, USA) under 0.6 MPa of NH_3_, where the ball-milling was performed at room temperature by using a planetary ball-mill apparatus (P5, Fritsch, Idar-Oberstein, Germany) with 250 rpm for 10 h. The purity of synthesized Mg(NH_2_)_2_ was evaluated by the weight loss with thermal decomposition (NH_3_ desorption) to 500 °C and estimated to be about 95%. Here, the synthesized Mg(NH_2_)_2_ possesses low crystallinity like nano-structural or amorphous phase because the crystalline size is decreased by the mechanical energy applied during the ball-milling process. To know the effect of starting crystalline size, LiH was pre-treated by the ball-milling (P7, Fritsch, Idar-Oberstein, Germany) under 1.0 MPa of H_2_ for 10 h and 34 h. The pristine or milled LiH was mixed with Mg(NH_2_)_2_ by the ball-milling under 1.0 MPa of H_2_ for 2, 20, and 40 h, where the molar ratio of Li and Mg was 8:3 (73:27 mol%). In addition, the catalyzed samples were also prepared, in which 0.54 mol% TiCl_3_ (98%) was added into the above mixing process for 20 h. The amount of TiCl_3_ was chosen to be 2 mol% for Mg(NH_2_)_2_ based on previous reports for the LiH-LiNH_2_ system [27,29]. For all the ball-milling process, 30 min interval is taken into every 1 h milling. The dehydrogenation of the samples was carried out at 200 °C for 24 h under dynamic vacuum condition. For the rehydrogenation, various procedures were performed as follows (see Figure S1). As the conventional thermochemical rehydrogenation, the dehydrogenated sample was heat-treated at 200 °C for 12 h under 10.0 MPa of H_2_. In addition, the rehydrogenated samples were ball-milled at room temperature under 1 MPa of H_2_ for 20 h, at 100 °C under 10 MPa of H_2_ for 5 h, and at −79 °C under 1 MPa of H_2_ for 2 h, where the products were named rehy + mill@RT, rehy + mill@100 °C, and rehy + mill@ −79 °C, respectively. For the ball-milling at −79 °C, the milling pot was cooled by the jacket filled with dry ice. Furthermore, the rehydrogenation was performed by the mechanochemical reaction. The dehydrogenated sample was milled at room temperature under 6.0 MPa of H_2_ for 5 h by a vibrating ball-mill apparatus (RM-10, Seiwa Giken, Hiroshima, Japan) and at 150 °C under 15.0 MPa of H_2_ for 5 h by a high-pressure type of vibrating ball-mill apparatus (RM-40, Seiwa Giken, Hiroshima, Japan). These rehydrogenated samples are named mill-rehy@RT and mill-rehy@150 °C. All the sample treatment was performed in a glove box filled with purified Ar (MP-P60W, Miwa MFG, Ibaraki, Osaka, Japan) because the Li and Mg compounds are easily oxidized in the air.

To identify the solid materials and discuss the structural properties, X-ray diffraction (XRD) measurement (RINT-2100, CuKα radiation, Rigaku, Akishima, Japan) was performed, where the samples were covered by a polyimide sheet (Kapton^®^, Du Pont-Toray Co., Ltd., Chuo-ku, Tokyo, Japan) to avoid sample oxidation. The hydrogen desorption properties were evaluated by thermogravimetry (TG, TG8120, Rigaku, Akishima, Japan) and mass spectroscopy (MS, M-QA200TS, Anelva, Kawasaki, Japan), which are placed into a glove box to measure essential properties of the samples without the influence of oxidation. For the TG-MS measurements, He gas was flowed as a carrier gas, suggesting that the H_2_ partial pressure around the sample would be kept to low level. The heating rate was fixed to be 5 °C·min^−1^. To evaluate the reaction rate and determine the utilizable hydrogen amount at 150 °C, an isothermal TG experiment was carried out, where the heating rate from room temperature to 150 °C was 5 °C·min^−1^ and the total measurement time was 8 h.

## 4. Conclusions

In this work, the synthesis and rehydrogenation processes of the 8LiH-3Mg(NH_2_)_2_ system were investigated to achieve the target properties, 5.5 mass% of hydrogen storage capacity and 150 °C of operating temperature.

For the synthesis, the effects of pre-milling for LiH, milling time of mixing LiH with Mg(NH_2_)_2_, and TiCl_3_ catalyst were examined. The pre-treatment decreased the crystalline size of LiH and improved the hydrogen desorption properties of the 2 h milled 8LiH-3Mg(NH_2_)_2_ sample. However, the 8LiH-3Mg(NH_2_)_2_ samples milled for more than 20 h was not improved by the pre-treatment of LiH. In this case, the effect of pre-treatment of LiH was negligible. TiCl_3_ revealed no catalytic effect for the 8LiH-3Mg(NH_2_)_2_ system although it was effective for the LiH-LiNH_2_ system. Thus, the crystalline size and mixing state are important factors to realize the fast hydrogen desorption properties.

For the rehydrogenation, the thermochemical and mechanochemical processes were investigated. After the only thermochemical rehydrogenation, the reaction rate of hydrogen desorption was clearly slowed due to the crystallization, which might be caused by the poor contact of the regenerated LiH and Mg(NH_2_)_2_. The ball-milling process for the rehydrogenated sample can recover the low crystalline size and showed fast hydrogen desorption. The mechanochemical rehydrogenation by ball-milling the dehydrogenated sample under H_2_ atmosphere was also useful because the hydrogen desorption properties of the obtained sample were almost the same as the synthesized sample, where the higher temperature of more than 150 °C was required to complete the hydrogen absorption. The rehydrogenated samples, which were obtained by the thermochemical rehydrogenation plus ball-milling and mechanochemical rehydrogenation, can desorb more than 5.5 mass% of H_2_ at 150 °C.

From the experimental facts obtained in this work, it is demonstrated that the 8LiH-3Mg(NH_2_)_2_ system can be considered a potential hydrogen storage materials to achieve the NEDO target by preserving the suitable crystalline size and contact state of LiH and Mg(NH_2_)_2_. Here, since a mechanical process, such as ball-milling, might not be useful as a practical application, other moderate techniques to prevent crystallization and isolation should be developed ideally. In addition, the reaction rate of the system is still slow for practical use, suggesting that further improvement is required in future works.

## Supplementary Materials

Supplementary materials can be accessed at: http://www.mdpi.com/1996-1944/8/7/3896/s1.
