# Supplementary material for: Kinetic Modification on Hydrogen Desorption of Lithium Hydride and Magnesium Amide System
Source: Materials (Basel). 2015 Jun 29;8(7):3896–909. doi: 10.3390/ma8073896 (PMC5455667; doi:10.3390/ma8073896)
Supplement: Supplementary file 1 [file materials-08-03896-s001.pdf]

Supplementary Materials

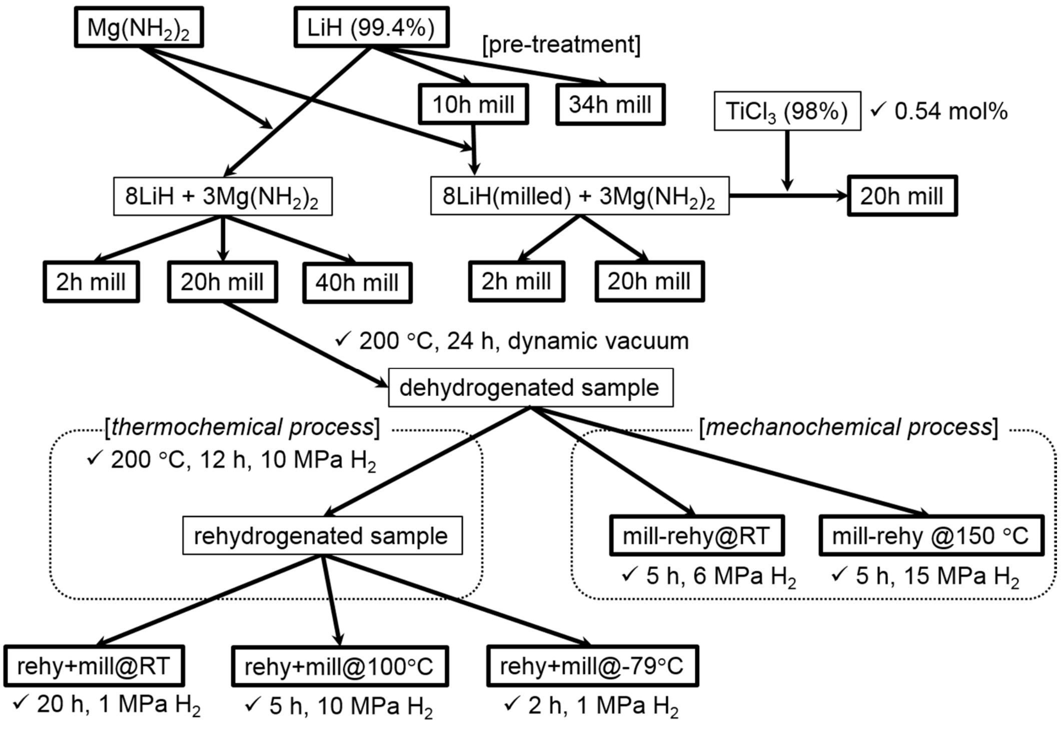

Figure S1. Flowchart for the sample preparation.

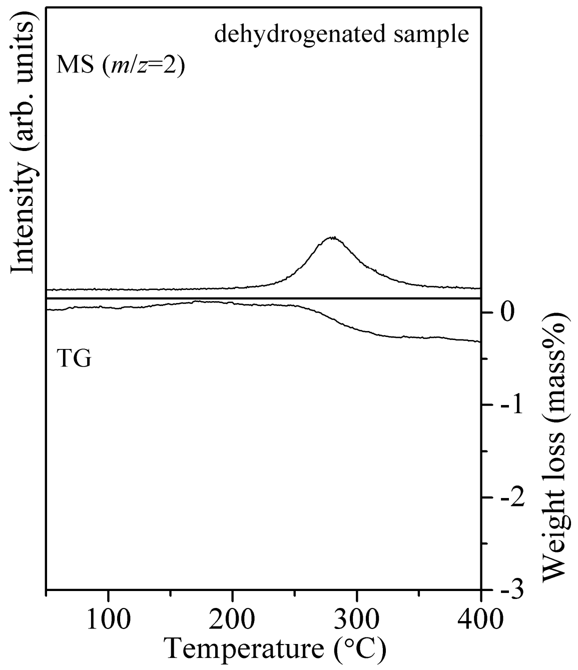

Figure S2. TG-MS profiles of the $8LiH\text{-}3Mg(NH_2)_2$ samples after the dehydrogenation at 200 °C under dynamic vacuum condition for 24 h.
